# Management of status epilepticus in pregnancy: a clinician survey

**DOI:** 10.1186/s42466-023-00295-z

**Published:** 2024-01-18

**Authors:** Dionne Swor, Pallavi Juneja, Charlotte Constantine, Catrin Mann, Felix Rosenow, Suzette LaRoche

**Affiliations:** 1 Department of Neurology, University of Toledo, Toledo, USA; 2 Department of Neurology, Columbia University, New York, USA; 3 Worcester Academy, Worcester, USA; 4 Department of Neurology, Epilepsy Center Frankfurt Rhine-Main, Goethe University Frankfurt, Frankfurt, Germany; 5 Department of Neurology, University of North Carolina, Chapel Hill, USA

**Keywords:** Status epilepticus in pregnancy, New onset status epilepticus in pregnancy, Women with epilepsy, Acute seizures, Anti-seizure medication

## Abstract

**Background:**

Status epilepticus in pregnancy (SEP) is rare and life-threatening for both mother and fetus. There are well-established guidelines for the management of women with epilepsy during pregnancy; however, there is little evidence guiding the management of SEP, leading to uncertainty among treating physicians. Therefore, this survey aims to investigate the real-world practices of physicians treating SEP to explore management approaches for improvements in care.

**Methods:**

An anonymous, electronic survey was created and distributed to neurointensivists and neurologists between September and December 2021.

**Results:**

One hundred physicians initiated the survey and 95 completed it in full: 87 (87%, 87/100) identified neurology as their primary specialty, 31 had subspecialty training in neurocritical care, and 48 had subspecialty training in epilepsy and/or clinical neurophysiology. Over half of the survey respondents (67%, 67/100) reported having participated in the management of SEP, with 48.9% (49/98) having done so in the past year. Most survey respondents (73%, 73/100) reported that their management approach to SEP is different than that of non-pregnant patients. Survey respondents were more likely to involve epilepsy consultants when treating SEP (58.5%, 58/99) and the vast majority involved Obstetrics/Maternal Fetal Medicine consultants (90.8%, 89/98). Survey respondents showed a clear preference for levetiracetam (89.7%, 87/97) in the treatment of benzodiazepine refractory status epilepticus followed by lacosamide (61%, 60/98) if an additional second line agent was needed. Valproate and phenobarbital were unlikely to be used. There was less agreement for the management of refractory and super-refractory SEP.

**Conclusions:**

Levetiracetam is the most frequently used anti-seizure medication (ASM) for benzodiazepine-refractory SEP. Survey participants tended to manage SEP differently than in non-pregnant patients including greater involvement of interdisciplinary teams as well as avoidance of ASMs associated with known teratogenicity.

## Background

SE is a neurologic emergency that occurs in up to 41 per 100,000 people and has an associated 30-day mortality rate of 10–27% [1–4]. SE in the general population is well studied, with established guidelines for management and treatment [5–9]. SEP, however, is not well studied. Prior randomized controlled studies including the Established Status Epilepticus Treatment Trial (ESETT) have excluded pregnant patients [6], leading to the lack of evidence for treatment of SEP. The existing body of literature assessing SEP is predominantly comprised of individual case reports and case series [10–12]. A recent literature review of SEP management revealed only 16 articles with a total of 39 pregnant women with SE [12]. The few cohort studies on this topic focus on women with a history of epilepsy and report varied incidence rates of SEP ranging from 1.3 to 8.5% [13, 14]. Patients with well-controlled seizures prior to pregnancy have a lower incidence of SEP, while patients who are poorly controlled are at increased risk of developing SEP [15, 16]. Less is known about the incidence of new onset status epilepticus in pregnancy (NOSEP) due to the variety of underlying pathologies. Though eclampsia with an incidence rate of 2/10,000 pregnancies is likely the predominant cause of NOSEP [17].

Seizure is the most common major neurologic complication in pregnancy [13]. When assessing causes and risk factors for SEP, it is important to distinguish between women with NOSEP and women with preexisting epilepsy who develop SEP. The former is often attributed to neurological conditions associated with pregnancy (i.e. cerebral venous thrombosis, reversible cerebral vasoconstriction syndrome, posterior reversible encephalopathy syndrome) and direct complications of pregnancy (i.e. eclampsia) [10–12, 18]. In pregnant women with a history of epilepsy, decreased ASM levels contribute to the risk of seizures if not carefully monitored and dose adjusted. Results from the Maternal Outcomes and Neurodevelopmental Effects of Anti-Epileptic Drug (MONEAD) study revealed that several commonly used ASMs, such as lamotrigine, levetiracetam, carbamazepine, and lacosamide had up to a 56% decrease in concentration during pregnancy [19].

Convulsive SEP is life-threatening to both mother and fetus as a result of blunt trauma, hypoxemia, and decreased blood flow to the placenta leading to fetal distress [20]. Therefore, the treatment of SEP requires prompt recognition, and involves unique management considerations that address the health of both the mother and the fetus. Practice parameters from the American Academy of Neurology [14] and the International League Against Epilepsy [21] help guide the management of women with epilepsy during pregnancy, but there is limited evidence to inform acute management of SEP. The most significant challenge is choosing a safe and effective ASM. Several first and second line ASMs used for the treatment of SE carry an increased risk of teratogenicity and adverse neurodevelopmental outcomes when used as maintenance therapy in pregnancy, particularly during the first trimester, which may influence how physicians acutely manage SEP [15, 22]. To help shed light on this challenge we developed a nationwide survey of neurologists and neurointensivists with the aim of understanding physician experience and management approach to SEP.

## Methods

### Survey creation and distribution

A 28-question survey was generated and distributed through the Research Electronic Data Capture (REDCap) Clinical and Translational Science Institute of the Wake Forest School of Medicine between September and December 2021. The survey was distributed to physician members of the Neurocritical Care Society (NCS) via website, the Critical Care EEG Monitoring Research Consortium (CCREMC) via email listserv, the American Epilepsy Society (AES) and the American Clinical Neurophysiology Society (ACNS) via newsletter. In addition, the survey was posted on social media groups targeted to neurologists and neurointensivists on Facebook, Twitter, and WhatsApp. Participation in the survey was voluntary and anonymous. The study was reviewed by the Institutional Review Board of Wake Forest University and received exempt approval.

### Statistical analysis

Descriptive analysis was performed to delineate the demographics and responses of survey participants. The results of the survey were presented as counts (percentage %). Univariate analysis of non-parametric data was conducted using Chi-Square test. R v4.2.0 (R Foundation for Statistical Computing, Vienna, Austria) was used to conduct statistical analysis.

## Results

### ***Demographics of survey respondents (***Table 1***)***

**Table 1:** Characteristics of survey respondents

*Access survey (n = 100)*	
Website	5
Email	29
Social media	47
Messenger app	17
Other	2
*Years in practice (n = 100)*	
In training	5
< 2 years	18
2–5 years	32
5–10 years	25
> 10 years	20
*Primary specialty (n = 100)*	
Neurology	87
Internal medicine	2
Anesthesia	1
Emergency medicine	4
Neurosurgery	1
Other	5
Subspecialty training*	
Neurocritical care	31
Critical care (non-neuro)	5
Epilepsy/neurophysiology	48
Vascular neurology	4
Other	25
*Practice type (n = 100)*	
Adults	90
Pediatrics	3
Both adult and pediatrics	7
*Practice setting (n = 100)*	
Academic/tertiary	89
Community medical center	7
Private practice	4
*Practice has affiliation with birthing center/performs deliveries (n = 99)*	
Yes	96
No	3
*Practice location (n = 95)*	
Northeast	27
Southeast	29
Midwest	16
Southwest	7
West	13
Outside of U.S.	3

*Multiple selections were allowed

The survey link was opened by 145 people, 100 (68.9%) answered at least one survey question and 95 answered the survey in full. Social media (47%, 47/100) was the most common vehicle for respondents to access the survey, followed by email (29%, 29/100), WhatsApp (17%, 17/100), and website (5%, 5/100). Eighty-seven (87%, 87/100) respondents identified neurology as their primary specialty. Of the 13 respondents identifying their primary specialty outside of neurology, 4 had neurocritical care fellowship training, 2 had critical care training, 2 had epilepsy training and 1 had neurosurgical training. Subspecialty training in neurocritical care was reported by 31, critical care 5, epilepsy and/or clinical neurophysiology 48, and dual neurocritical care and epilepsy/clinical neurophysiology training in 6. Practice setting was largely an academic or tertiary referral center and primarily focused on treatment of adult patients.

### Experience managing status epilepticus and status epilepticus in pregnancy

Nearly all survey respondents (90%, 90/100) reported experience in the management of SE in the general population, with 70% (70/100) routinely managing 10 or more patients with SE per year. Most had access to continuous EEG monitoring (93/100), but only a minority routinely consulted an epileptologist for assistance in management (38/100).

Sixty-seven of 100 survey respondents reported having managed at least one case of SEP, with 49% (48/98) occurring within the past year (Table 2). Common reported etiologies of SEP included a prior diagnosis of epilepsy in half of the patients (50), and NOSEP in the other half. Identified etiologies included eclampsia (41), vascular lesion (29), tumor/mass lesion (12), toxic/metabolic derangement (11), and meningitis/encephalitis (10). The survey did not assess for differences in the etiologies of SEP vs NOSEP.

In general, 73% (73/100) of survey respondents reported that their approach to the management of SEP is different than in a non-pregnant patient (Table 2) with no significant difference among clinicians with and without subspecialty training in epilepsy (*p *= 0.971). Survey respondents were more likely to involve epilepsy consultants when treating SEP, 58.5% (58/99) as opposed to 38% (38/100) for SE management in non-pregnant patients. Nearly all survey respondents involved Obstetrics/Maternal Fetal Medicine consultants, 90.8% (89/98). There was no difference between clinicians with epilepsy training and those without epilepsy training in regard to involvement of epilepsy (*p *= 0.125) or OB/GYN (*p *= 0.537) consultants. Survey respondents reported avoiding ASMs associated with increased risk of major congenital malformations, including, valproate (85), phenobarbital (51), and phenytoin (42) (Table 2). Gestational age was a driving factor in clinical decision making in 79% (79/100).

**Table 2:** Management survey questions of status epilepticus in pregnancy

*Managed status epilepticus in a pregnant patient (n = 100)*	
Yes	67
No	33
*Number of pregnant patient with SE managed in the past year (n = 98)*	
0	50
1	27
2–5	20
> 5	1
Etiology of status epilepticus in pregnancy that respondents have Managed*	
Eclampsia	41
Prior diagnosis of epilepsy	50
Vascular lesion	29
Tumor/mass lesion	12
Meningitis/encephalitis	10
Autoimmune/paraneoplastic	8
Traumatic brain injury	8
Toxic/metabolic	11
Other	3
Unknown	4
Never managed	27
*Is your general approach for SE treatment different in pregnant patients (n = 100)*	
Yes	73
No	27
*Epilepsy consult for management of SE in pregnant patient (n = 99)*	
Always	35
Usually	6
Sometimes	17
Never	8
Would like to, but no epileptologist available	4
Not applicable/I am an epileptologist	29
*Obstetrics/maternal fetal medicine consult (n = 98)*	
Always	80
Usually	6
Sometimes	3
Never	1
3rd trimester only	2
Not applicable	6
Anti-seizure medications avoided in treatment of SE in pregnancy*	
Levetiracetam	1
Fos-phenytoin/phenytoin	42
Valproate	85
Lacosamide	3
Phenobarbital	51
Other	2
*Does gestational age factor into your treatment decision (n = 100)*	
Yes	79
No	21

*Multiple selections were allowed

*Hypothetical Clinical Scenario of NOSEP* A 26-year-old pregnant person, who is in their second trimester of pregnancy with no additional past medical history presents to the emergency department (ED) with complaints of headache and a first-time seizure. Imaging reveals a superior sagittal sinus thrombosis and adjacent 7 mm right frontal-parietal intracerebral hemorrhage with associated edema. Lab work is significant for mildly elevated lactate and white blood cell count, normal metabolic panel, and negative drug toxicology screen. While in the ED, the patient has a witnessed clinical seizure lasting > 5 min that is refractory to an appropriate dose of benzodiazepine. You are asked for further seizure management recommendations.

Survey respondents were asked for their preferred management recommendations for NOSEP in the above clinical scenario (Table 3). Levetiracetam was chosen by 89.7% (87/97) of which only 27.8% (27/97) reported this choice as different from what they would typically choose in a non-pregnant patient in SE. When asked for their choice of the next second line ASM, 61.2% (60/98) of survey respondents chose lacosamide followed by phenytoin 20.4% (20/98). A larger percentage 48.9% (48/98) reported this second choice ASM as different from what they would typically choose in a non-pregnant patient. There was no difference in first (*p *= 0.486) or second choice (*p *= 0.521) ASMs among neurologists with different subspecialties.

**Table 3:** Survey response to clinical scenario of SEP management

*Which second line anti-seizure medication would you choose? (n = 97)*	
Levetiracetam	87
Fos-phenytoin/phenytoin	4
Valproate	0
Lacosamide	0
Phenobarbital	0
Magnesium	6
Other	0
*Is this choice different from your typical choice?*	
Yes	27
No	70
*If a second, second line anti-seizure medication is needed what would you choose? (n = 98)*	
Levetiracetam	8
Fos-phenytoin/phenytoin	20
Valproate	3
Lacosamide	60
Phenobarbital	3
Magnesium	3
Other	1
*Is this choice different from your typical choice?*	
Yes	48
No	50
*What anesthetic drip do you choose to treat refractory SE in pregnancy? (n = 100)*	
Propofol	36
Midazolam	60
Ketamine	2
Pentobarbital	1
Sevoflurane	0
Other	1
*Is this choice different from your typical choice?*	
Yes	18
No	82
What alternative treatments for super refractory SE have you used in pregnant patients?*	
Therapeutic hypothermia	8
Ketogenic diet	7
High dose steroids	18
Plasma exchange	7
Trans-magnetic stimulation	2
Neurosurgical intervention	7
Other	10
None	63

*Multiple selections were allowed

When asked about intravenous (IV) anesthetics for treating refractory SEP, 60% (60/100) of survey respondents chose midazolam and 40% (40/100) chose propofol with only 18% (18/100) reporting this choice being different from their typical practice in a non-pregnant patient. Treatment of super refractory SEP was varied among survey respondents with high dose steroids (18%) being the most commonly chosen option.

## Discussion

This survey offers insight into the real-life-management of SEP where there is currently a paucity of data despite significant consequences for the health of pregnant women and the fetus [14, 21]. Despite lack of clear data, the majority of respondents reported that their general approach to the treatment of SEP is different to that of non-pregnant SE patients including increased likelihood of employing an interdisciplinary team approach.

SEP can be divided into two broad categories: women with a prior history of epilepsy and NOSEP. Patients presenting with NOSEP, can be further subdivided by etiology into eclampsia and other acute symptomatic etiologies. For the treatment of seizures in eclampsia, magnesium is well established as the ASM of choice and has been shown to be superior to diazepam in controlling recurrent seizures with decreased maternal morbidity in comparison to the use of phenytoin [23, 24]. However, when given a hypothetical clinical scenario of NOSEP and asked to choose an ASM following benzodiazepine failure, survey respondents almost unanimously chose levetiracetam (89.6%) with magnesium chosen by only 6 percent. This likely suggests that while magnesium may be the ASM of choice for eclampsia, it is not necessarily considered the best choice for acute symptomatic etiologies unless there is concomitant diagnosis of eclampsia. The choice of levetiracetam is not surprising as it is recommended in guidelines for secondline treatment of SE [5] and it has been shown to be equally efficacious to valproate and phenytoin in a randomized controlled trial [6].

If an additional second line ASM was needed in the aforementioned clinical scenario, most survey respondents preferred lacosamide (61%), with nearly half reporting this choice as different from what they would typically choose in non-pregnant patients. Lacosamide, however, is not currently recommended as second line treatment for SE following benzodiazepine failure and has limited, albeit growing evidence for its use in SE management [25–27]. Furthermore, only small case series evaluating safety of lacosamide in pregnancy have been published. It is important to recognize that the absence of data demonstrating teratogenicity for new medications like lacosamide is not a proof of safety [28]. While animal data does not always predict human data, lacosamide increased embryofetal and perinatal mortality and growth deficits in rats following administration during pregnancy [29].

Balancing the risk of seizures against known teratogenicity associated with ASMs is central to the management of SEP. Chronic ASM exposure is associated with a higher risk of major congenital malformations and adverse neurodevelopmental outcomes [15, 22]. Valproate carries the highest risk of major congenital malformations followed by phenobarbital and fos/phenytoin, while lamotrigine and levetiracetam are associated with the lowest risk [15, 30, 31]. Accordingly, survey respondents tended to avoid ASMs associated with higher rates of major congenital malformations with valproate followed by phenobarbital and phenytoin reported as medications least likely to be used in pregnant patients.

The majority of survey respondents (79%) considered gestational age when selecting treatment options for SEP, which suggests variability of ASM choices at various time points of pregnancy. Most pregnancy registries prospectively assess risk of ASM exposure starting with the first trimester [15, 32]. ASM exposure after the first trimester of pregnancy is not associated with an increased risk of major congenital malformations [33] due to the completion of organogenesis within the first trimester. However, prenatal ASM exposure during the second and third trimester may lead to adverse neurodevelopmental effects [22], low birth weight or poor neonatal adaptation [34]. The benefits of treatment during this time frame may outweigh the risk, particularly if the exposure is brief. Therefore, it may be reasonable to consider a short course of phenytoin, valproate, or phenobarbital outside of the first trimester, although this needs additional study.

A recent literature review on SEP proposed a treatment strategy for SE management in the pregnant patient with levetiracetam recommended as the initial second line ASM followed by fos/phenytoin and then valproate only if the prior ASM’s fail to control SE [12]. The proposed protocol does not mention the use of lacosamide, which was the second most commonly selected second line ASM chosen in our survey. This is likely due to the lack of data for the use of lacosamide during pregnancy and in the management of SE, in addition, many of the case reports sited in the review pre-dated FDA approval of lacosamide. A separate review proposed a management strategy according to the different clinical manifestations of SEP, discerning between women with epilepsy and NOSEP [18]. This proposed management algorithm follows the standard SE treatment guidelines with the exception of recommending avoidance of valproate in all presentations of SEP and to identify and treat the underlying cause of NOSEP [18]. Both treatment proposals advise following established treatment guidelines for SE with caution in using valproate and no specific mention of alternate ASM’s such as lacosamide.

In regard to medication management for refractory and super-refractory SEP, survey responses were varied but generally aligned with current guideline recommendations for SE in non-pregnant patients and most respondents reported no deviation from their typical treatment management approach. Unlike the literature on ASMs, the literature on the effect of benzodiazepine use on fetal outcomes is divided [35, 36]. The majority of studies evaluated chronic use of benzodiazepines as anxiolytics, which is markedly different than use for acute management of SEP, either as first line treatment or as a continuous IV infusion for refractory SEP. One study of short-term diazepam use, defined as 3 weeks, found no increased teratogenic risk to the fetus [37]. For SEP refractory to IV anesthetics, steroids was the most common selection, however there was no majority among choices, likely because this is an uncommon clinical situation and most respondents reported they had not used any of the options in their own practice. One case report described two patients who had resolution of super-refractory SEP following delivery of the infant suggesting a component of autoimmune or hormone fluctuations as an underlying cause of the SE [38].

Termination of pregnancy is a controversial topic and there are no established standards on when termination of pregnancy should be considered in this situation. Gestational age plays a key role in decision-making, and beyond 34 weeks' gestation, a timely delivery should usually be sought. Traditional obstetrical teaching advises, that when managing seizures or SE in a pregnant patient every effort should be made to stabilize the mother and resuscitate the fetus prior to deciding about delivery [39]. Severe eclampsia (which includes complications of refractory SE) should always lead to rapid delivery in the last trimester of pregnancy as this is also a treatment component of eclampsia itself [40].

There are several limitations to this study. The main drawback of the study is the survey design, which is subject to recall and selection bias. This is particularly relevant in our study where the physician’s subspecialty of neurocritical care versus epilepsy may have skewed the selection of ASM and management of SEP based on personal preference and experience. While most survey participants were neurologists not all neurointensivists are neurologists which may have influenced management choices. In addition to these challenges, the sample size was small despite being distributed to several professional societies comprised of members whose clinical interests include treatment of SE. The survey was distributed through professional society’s websites, newsletters, email lists, and social media which made determining a true response rate near impossible. Approximately half of the survey participants accessed the survey through social media outlets including Twitter, which is an open domain, and therefore, it is possible that non-clinicians completed the survey. Lastly, because identifying information was not collected, it is possible that a respondent could have completed the survey more than once.

## Conclusion

Management of SEP is clinically complex and requires clinicians to consider the health outcomes of both the pregnant person and the fetus. Thus, physicians are more likely to involve interdisciplinary teams in the management of SEP. In general, survey respondents adhered to established guidelines for SE management, with levetiracetam being the preferred treatment for benzodiazepine refractory SEP. In a departure from guideline recommendations, survey respondents preferred lacosamide when an additional ASM was needed despite any clear evidence for its use in SE or substantial research on its risks in pregnancy. Furthermore, it is uncertain whether the benefit of short-term exposure to ASMs with known teratogenicity may outweigh risk, particularly if used outside of the first trimester and when other treatment options have failed. More research is needed to determine optimal treatment strategies of SEP.

## Data Availability

All data generated and analyzed during this study are available from the corresponding author on reasonable request.

## Abbreviations

SEP: Status epilepticus in pregnancy
NOSEP: New onset status epilepticus in pregnancy
SE: Status epilepticus
ASM: Anti seizure medication
